# Light-induced retinal ganglion cell damage in vivo involves Dexras1

**Published:** 2011-01-13

**Authors:** Aimin Sang, Yanyan Cheng, Hong Lu, Doudou Chen, Ruifang Gao, Aiguo Shen

**Affiliations:** 1 Department of Ophthalmology, the Affiliated Hospital of Nantong University, Medical College, Nantong University, Nantong, China; 2 Jiangsu Province Key Laboratory of Neuroregeneration, Nantong University, Nantong, China

## Abstract

**Purpose:**

Light-induced retinal degeneration is a vision-threatening retinal disease. Light can damage not only photoreceptor cells but also retinal ganglion cells (RGCs). This study was aimed to observe the spatiotemporal expression of dexamethasone-induced Ras protein 1 (Dexras1) and document the effect of Dexras1 on RGC damage after light exposure.

**Methods:**

Adult Sprague-Dawley rats were exposed to bright white light for 2 h. Reverse transcriptase-PCR (RT–PCR) and western blot analysis were used to analyze mRNA and protein expression of Dexras1. The spatial distribution of Dexras1 and outer nuclear layer (ONL) thickness were evaluated by immunohistochemistry. Immunoﬂuorescence was performed to observe the colocalization of Dexras1. In addition, cell apoptosis in this model was measured using terminal deoxynucleotidyl transferase deoxyuridine triphosphate (dUTP) nick end labeling (TUNEL). Finally, the effect of systemic administration of nitric oxide synthase (NOS) inhibitor on the retina was investigated by western blot analysis and immunoﬂuorescence.

**Results:**

*Dexras1* expression increased at 6 h and reached the peak at 1 day, gradually recovering to the baseline level at 7 days after light exposure. Dexras1 immunoreactivity was detected in RGCs and colabeled with cleaved caspase-3 after light exposure, whereas cleaved caspase-3 immunoreactivity was undetectable in the ONL. However, immunohistochemistry demonstrated that the ONL thickness decreased after light exposure and TUNEL revealed that photoreceptor cell apoptosis also occurred. In addition, the ternary complex of Dexras1, neuronal NOS (nNOS), and the C-terminal PSD95/DLG/ZO-1 ligand of nNOS was observed in RGCs. Administration of NOS inhibitor decreased the expression of cleaved caspase-3 and Dexras1.

**Conclusions:**

Exposure to light caused the transient high expression of Dexras1, which was colabeled with apoptotic marker, nNOS, and the C-terminal PSD95/DLG/ZO-1 ligand of nNOS in RGCs. Administration of the NOS inhibitor prevented RGC apoptosis by decreasing cleaved caspase-3 and Dexras1 expression. Dexras1-mediated RGC damage appears to act through activation of nNOS in this model.

## Introduction

The human retina is protected from shorter wavelength radiation by the cornea and lens, which absorb ultraviolet light below 400 nm [1]. The retina is therefore exposed mainly to the “visible component” of light. Light is an additional factor that can lead to retinal damage. Light-induced retinal damage has been used as a model to study retinal degeneration [2-5], which has an important feature: photoreceptor cell death by apoptosis [6]. However, ganglion cells, which are densely laden with mitochondria and contain photopigment, might also be susceptible to excessive light injury [7]. Recent findings show that excessive light can negatively affect ganglion cell survival directly in vivo and in vitro [7,8].

The molecular mechanism of light-induced retinal ganglion cell (RGC) damage remains unclear. Nitric oxide (NO) plays critical roles in the eye at various physiologic levels [9] but leads to neuronal cell death when produced in excess [10]. NO is synthesized by a group of isoenzymes known as NO synthase (NOS), which consists of three members: neuronal NOS (nNOS), inducible NOS, and endothelial NOS [11,12]. In the light-induced retinal degeneration model, the results reveal no detectable increase in either inducible NOS or endothelial NOS expression; only nNOS expression increases significantly after light damage [13]. nNOS, a calcium (Ca^2+^)/calmodulin-dependent enzyme, is reported to be induced in many pathological processes, including RGC apoptosis [14]. One of the regulators of nNOS is the N-methyl-D-aspartate receptor (NMDAR), an excitatory glutamate receptor consisting of N-methyl-D-aspartate receptor 1 (NMDAR1 or NR1) and N-methyl-D-aspartate receptor 2 (NMDAR2 or NR2) subunits that is targeted to excitatory synapses, where it functions in neural plasticity [15]. In neurons, stimulation of NMDAR activates nNOS, leading to S-nitrosylation and activation of dexamethasone-induced Ras protein 1 (Dexras1) [16].

Dexras1 is a 30 kDa G protein in the Ras subfamily whose discovery was based on its pronounced inducibility by the glucocorticoid dexamethasone. It shares about 35% homology with the Ras subfamily of proteins and contains all of the conserved domains of typical guanosine triphosphatase (GTPases). Similar to other members of the Ras subfamily, Dexras1 possesses four highly conserved motifs for GTP-binding and hydrolysis, an effector loop that mediates protein–protein interactions, and a membrane-targeting CAAX (C is a cysteine, the two A residues are aliphatic amino acids and the X can be one of several amino acids) box, which serves as a consensus site for isoprenylation. Unlike conventional GTPases, Dexras1 contains an extended 7 kDa C-terminal cationic domain [17]. Dexras1 was identified as a binding partner for the C-terminal PSD95/DLG/ZO-1 ligand of nNOS (CAPON), a scaffolding protein that interacted with nNOS [18]. The existence of ternary complexes of Dexras1, nNOS, and CAPON was confirmed in the brain and spinal cord [19,20]. Several studies indicated that Dexras1 is a downstream physiologic target of nNOS-mediated signaling [20,21].

The present investigations were designed to detect the temporal and spatial patterns of Dexras1 expression, as well as its cellular localization, possible role in RGC death, and association with nNOS and CAPON after light exposure. Finally, we demonstrated the effect of the NOS inhibitor on cleaved caspase-3 and Dexras1 expression.

## Methods

### Animals

Adult Sprague-Dawley rats, either sex (Department of Animal Center, Medical College, Nantong University), were used in our experiments. Rats weighing 180–220 g were kept on a 12 h:12 h light-dark schedule and given food and water in a pathogen-free area. All experimental procedures were performed in accordance with the Association for Research in Vision and Ophthalmology Statement for the Use of Animals in Ophthalmic and Vision Research.

### Light damage

Before light exposure, rats were dark adapted for 1 day. After pupil dilation with compound tropicamide (Santen Pharmaceutical, Osaka, Japan), dark-adapted rats were placed in cages and exposed to white light (16,000 lx) for as long as 2 h, beginning at 9 AM During light exposure, the room temperature was kept at 24 °C and the animals had free access to food and water. After light exposure, all rats were returned to darkness.

### Intraperitoneal injections

Rats were injected intraperitoneally with the following: the nonspecific NOS inhibitor N^G^-nitro-L-arginine methyl ester (L-NAME), 100 mg/kg (Sigma, St. Louis, MO) in PBS (145 mM NaCl, 24 mM Na_2_HPO_4_, 1.2 mM NaH_2_PO_4_) or PBS alone as a control. All intraperitoneal injections were administered immediately after light exposure.

### *Total RNA isolation and* s*emiquantitative RT–PCR*

Total RNA was extracted from retinas with TRIzol (Invitrogen, Paisley, UK). RNA was reverse-transcribed using the moloney murine leukemia virus (M-MLV) reverse transcriptase PCR system (RT–PCR; Promega, Southampton, UK). cDNA was equalized in an amplification reaction with *Dexras1* primers (forward, 5′-GCT TAG ACA ACC GCG ACT CCT TC-3′; reverse, 5′-CTC AAT CTC CCG CTG CTC CAC TT-3′), yielding a 173 bp product. PCR amplification was performed with an initial denaturing step at 94 °C for 3 min, then 30 cycles at 94 °C for 45 s, 59 °C for 45 s, 72 °C for 1 min, and a further extension at 72 °C for 7 min.

### Western blot analysis

Total protein was obtained by lysing in a buffer containing 1 M Tris-HCl pH 7.5, 1% Triton X-100, 1% NP-40 (nonidet p-40), 10% sodium dodecyl sulfate, 0.5% sodium deoxycholate, 0.5 M EDTA, 10 μg/ml leupeptin, 10 μg/ml aprotinin, and 1 mM phenylmethanesulfonyl fluoride or phenylmethylsulfonyl fluoride. Protein was separated with sodium dodecyl sulfate- PAGE and transferred to polyvinylidine difluoride filter membranes (Millipore, Bedford, MA). The membranes were incubated overnight with Dexras1 (Santa Cruz Biotechnology, Santa Cruz, CA) or cleaved caspase-3 (Cell Signaling Technology, Danvers, MA) at 4 °C. Finally, appropriate secondary antibodies conjugated to horseradish peroxidase were subsequently added and the films were visualized using an enhanced chemiluminescence system (Pierce Company, Minneapolis, MN).

### Sections and immunohistochemistry

After adult rats were anesthetized and perfused, the superior conjunctiva was sutured with 8.0 vicryl as a reference that provided orientation [22]. The eyes were fixed in 4% paraformaldehyde solution, followed by immersion in sucrose solution for cryoprotection. Then the tissues were embedded in opti-mum cutting temperature (OCT) compound and 8 μm frozen sections were prepared. The superior hemisphere along the vertical meridian was chosen in this experiment. Sections were blocked with 10% normal serum blocking solution and incubated overnight at 4 °C with Dexras1. Then, slides were incubated in biotinylated secondary antibody, followed by incubation in the complex avidin-biotin-peroxidase. In negative control sections, the primary antibody was substituted by PBS. The reaction product was revealed by 0.02% diaminobenzidine tetrahydrochloride.

### Terminal deoxynucleotidyl transferase-mediated dUTP nick end labeling

TUNEL staining was performed using the DeadEnd™ Fluorometric TUNEL System (Promega, Madison, WI). Frozen tissue sections were rinsed in PBS and treated with 1% Triton X-100 in PBS for 2 min on ice. Slides were equilibrated with equilibration buffer and then incubated for 60 min at 37 °C with recombinant terminal deoxynucleotidyl transferase (rTdT) incubation buffer. The negative control sections were incubated with control incubation buffer without the rTdT enzyme. The slides were analyzed using a Leica TCS SP5 confocal microscope (Germany).

### Immunofluorescence

The sections were blocked with 1% BSA (BSA) to avoid unspecific staining. Then, sections were incubated overnight at 4 °C with Dexras1, CAPON (Santa Cruz Biotechnology, Santa Cruz, CA), nNOS (Abcam, Cambridge, MA), cleaved caspase-3, and neuronal nuclei (NeuN; Chemicon International, Temecula, CA). After washing in PBS, a mixture of fluorescein isothiocyanate- and Cy3-conjugated secondary antibodies was added. The images were captured by Leica fluorescence microscope (Germany).

### Statistical analysis

All values were expressed as means±standard error of mean (SEM). The statistical significance of difference between groups was determined by one-way ANOVA (ANOVA) followed by Tukey’s post hoc multiple comparison tests. p<0.05 was considered significant.

## Results

### Changes in mRNA and protein expression for Dexras1 after light exposure

**Figure 1:**
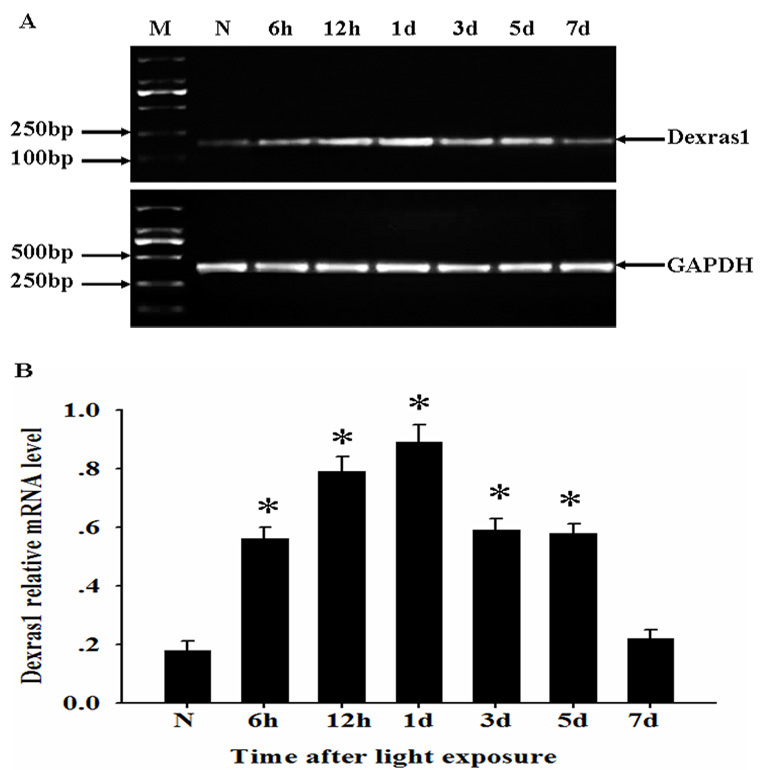
*Dexras1* mRNA level upregulated in the retina after light exposure. **A**: Total RNA was extracted from normal and injured retina at various times after light exposure, then assessed by reverse transcriptase (RT)-PCR. mRNA for *Dexras1* increased at 6 h after light exposure and reached the peak at 1 day, gradually recovering to the baseline level at 7 days. **B**: Relative mRNA level represented a ratio between the amount of target gene and amount of endogenous *GAPDH* control. Groups marked with an asterisk were significantly different from the normal group. The data are means±SEM (n=3, *p<0.05).

**Figure 2:**
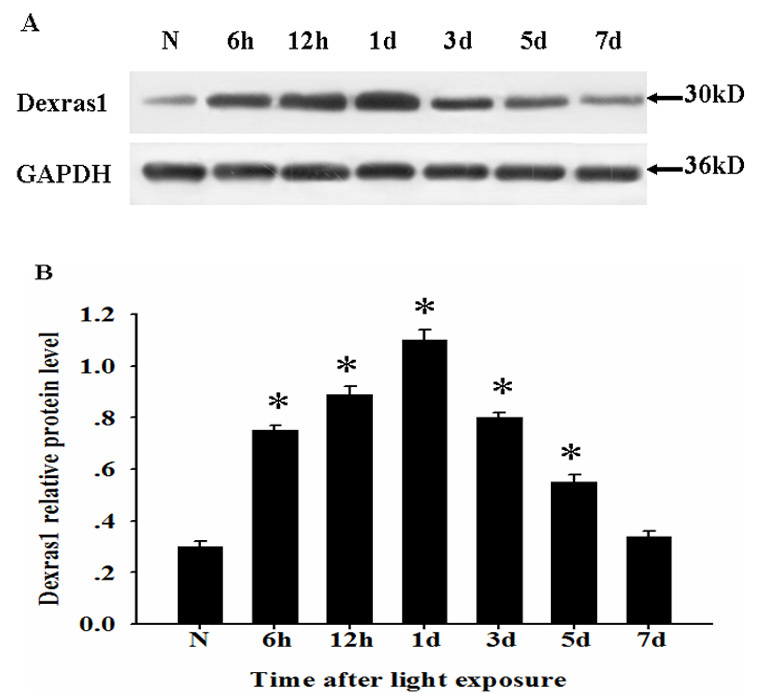
Dexras1 protein level upregulated in the retina after light exposure. **A:** Total protein was extracted from normal and injured retinas at various times after light exposure, then assessed by western blot analysis. **B:** Relative protein level represented a ratio between the amount of target gene and amount of endogenous glyceraldehyde-3-phosphate dehydrogenase (GAPDH) control. Groups marked with an asterisk were significantly different from the normal group. The data are means±standard error of mean (SEM; n=5, *p<0.05).

Semiquantitative RT–PCR was performed to investigate the temporal pattern of *Dexras1* mRNA expression after light exposure. We found that mRNA for *Dexras1* was relatively low in the normal retina, increased beginning at 6 h after light exposure (p<0.05) and reached the peak at 1 day, gradually recovering to the baseline level at 7 days (Figure 1A,B). In comparison with RT–PCR analysis, we performed western blot analysis to detect Dexras1 protein expression. The trends of Dexras1 protein expression were similar to that of *Dexras1* mRNA expression. At 6 h after light exposure, Dexras1 protein expression increased, reached the peak at 1 day, and then gradually declined from 3 to 7 days (Figure 2A,B).

### Distribution of Dexras1 and change of outer nuclear layer thickness after light exposure

**Figure 3:**
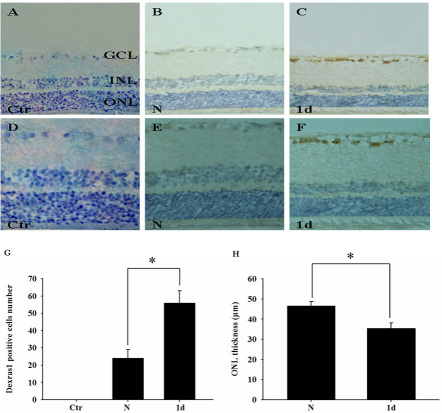
The distribution of Dexras1 expression and the change in outer nuclear layer thickness after light exposure. Slides were examined at 200× or 400× magnification on a Leica light microscope. Dexras1 expression and outer nuclear layer (ONL) thickness were observed in sections of **A:** negative control retina (primary antibody was substituted by PBS), **B:** normal retina, and **C:** injured retina at 1 day after light exposure by immunohistochemistry. **D**-**F:** Magnified images for **A**-**C**, respectively, are represented. **G:** Quantitative results for numbers of Dexras1 positive cells are at a lower magnification. **H:** Quantitative analysis of ONL thickness between the normal retina and injured retina was conducted. Values are expressed as mean±SEM (n=3 animals, 6 eye samples from three animals for each group, *p<0.05). Abbreviations: GCL represents ganglion cell layer; INL represents inner nuclear layer; ONL represents outer nuclear layer.

Immunohistochemistry was performed to observe Dexras1 distribution and ONL thickness. Slides were examined at 200× or 400× magnification on a Leica light microscope. Figure 3D-F show the magnified images of Figure 3A-C, respectively. In control sections preincubated with PBS, the expression of Dexras1 was undetectable (Figure 3A,D). In the normal retina, Dexras1 immunoreactivity was relatively low in the ganglion cell layer (GCL; Figure 3B,E). At 1 day after light exposure, strong Dexras1 positive cells increased significantly in the GCL (Figure 3C, F) compared with the normal retina based on quantitative study (Figure 3G). Furthermore, we evaluated the change of ONL thickness after light exposure. In the injured retina, ONL thickness decreased significantly compared with the normal retina (Figure 3E,F) based on quantitative analysis (Figure 3H).

### Dexras1 colocalization with markers and TUNEL labeling after light exposure

**Figure 4:**
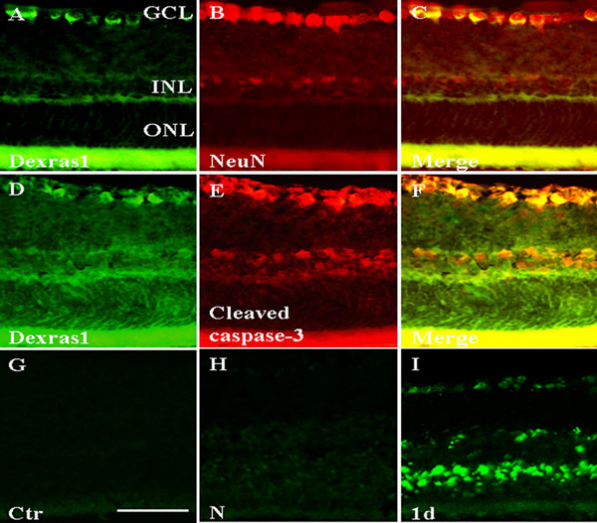
Dexras1 colocalization with markers and terminal deoxynucleotidyl transferase deoxyuridine triphosphate (dUTP) nick end labeling (TUNEL) labeling after light exposure. Immunoﬂuorescence was performed to observe Dexras1 colocalization in the retina at 1 day after light exposure. The neuronal nuclei (NeuN) was used here as the marker of ganglion cells. **A**-**C**: Dexras1 immunoreactivity overlapped with NeuN-positive neurons in the retina. **D**-**F**: Cleaved caspase-3 as an apoptotic marker was colocalized in Dexras1-positive cells in the retina. **C**, **F:** Merged images. Magnification, 400×. TUNEL results were observed in sections of **G**: control retina, **H**: the normal retina, and **I**: the injured retina at 1 day after light exposure. In the control and normal retina, there were no TUNEL-positive cells. In the injured retina at 1 day after light exposure, TUNEL-positive labeling was apparent in the ONL, INL, and GCL. The scale bar is 50 µm in **G**-**I**. Abbreviations: GCL represents ganglion cell layer; INL represents inner nuclear layer; ONL represents outer nuclear layer.

Immunoﬂuorescence was performed on cryosections of the retina at 1 day after light exposure. The labeling was examined using a Leica ﬂuorescence microscope at 400× magnification. The expression of Dexras1 in the retina was detected by the anti-Dexras1 primary antibody (Figure 4A). NeuN (a neuron marker) was used here as the marker of the ganglion cell (Figure 4B). Dexras1 immunoreactivity overlapped with NeuN-positive neurons (Figure 4C). Based on evidence that caspase-3 activity has a positive correlation with RGC apoptosis after light exposure [8], cleaved caspase-3 was used here as an apoptotic marker to investigate whether apoptosis occurred in Dexras1-positive cells. Colocalization of cleaved caspase-3 and Dexras1 demonstrated that cell apoptosis occurred in Dexras1-positive cells (Figure 4D-F). It was interesting to find that cleaved caspase-3 immunoreactivity was not detected in the ONL. With the objective of elucidating whether photoreceptor cell apoptosis occurred, we used TUNEL labeling to show cell apoptosis in our model. In the control and normal retina, there were no TUNEL-positive cells (Figure 4G,H). At 1 day after light exposure, TUNEL labeling was apparent in the ONL, inner nuclear layer (INL), and GCL (Figure 4I).

### Colocalization of Dexras1, nNOS, and CAPON after light exposure

**Figure 5:**
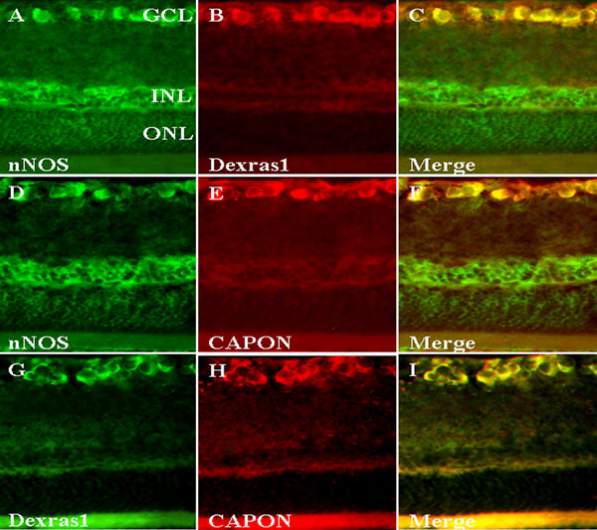
Colocalization of Dexras1, neuronal nitric oxide synthase (nNOS), and C-terminal PSD95/DLG/ZO-1 ligand of neuronal nitric oxide synthase (CAPON) after light exposure. Immunoﬂuorescence was performed to observe Dexras1 colocalization with nNOS and CAPON in the retina at 1 day after light exposure. **A-C**: nNOS immunoreactivity was colocalized in Dexras1-positive cells in the retina. **D-F**: nNOS immunoreactivity was colocalized in CAPON-positive cells in the retina. **G-I**: Dexras1 immunoreactivity was colocalized in CAPON-positive cells in the retina. **C**, **F**, **I**: Merged images. Magnification, 400×. Abbreviations: GCL represents ganglion cell layer; INL represents inner nuclear layer; ONL represents outer nuclear layer.

To investigate whether Dexras1 was associated with neuronal nitric oxide synthase (nNOS) and C-terminal PSD95/DLG/ZO-1 ligand of neuronal nitric oxide synthase (CAPON) during the pathological process after light exposure, immunofluorescence was used on sections of the retina at 1 day after light exposure. nNOS and CAPON immunoreactivity overlapped with NeuN-positive neurons (data not shown). Colocalization of nNOS and Dexras1 appeared in RGCs (Figure 5A-C). nNOS immunoreactivity could be colocalized with CAPON immunoreactivity in RGCs (Figure 5D-F). Dexras1 expression and CAPON expression were also colabeled in RGCs (Figure 5G-I). These observations showed that colocalization of Dexras1, nNOS, and CAPON was present in RGCs.

### Effects of NOS inhibitor N^G^-nitro-L-arginine methyl ester on the expression of cleaved caspase-3 and Dexras1

**Figure 6:**
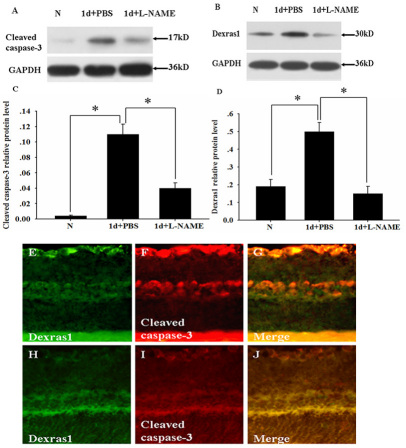
Inhibition of nitric oxide synthase (NOS) by L-NAME decreased the expression of cleaved caspase-3 and Dexras1. Adult rats were injected intraperitoneally with L-NAME or PBS as a control. At 1 day after light exposure, the injured retina was prepared. **A**-**B**: western blot analysis showed cleaved caspase-3 and Dexras1 expression in the normal retina, injured retina with PBS treated group, and injured retina with L-NAME treated group. **C**-**D**: Quantitative analysis of **A** and **B**, respectively. Relative protein level represented a ratio between the amount of target gene and amount of endogenous GAPDH control. Groups marked with an asterisk were significantly different from the control group. The data are means±SEM (n=5, *p<0.05). Immunofluorescence showed colabeling of Dexras1 and cleaved caspase-3 in the retina at 1 day after light exposure with **E**-**G**: the PBS treated group and **H**-**J**: the L-NAME treated group. **G**, **J**: Merged images. Magnification, 400×. Abbreviations: GCL represents ganglion cell layer; INL represents inner nuclear layer; ONL represents outer nuclear layer.

We next examined the effect of the administration of the NOS inhibitor L-NAME on the retina after light exposure. Adult rats were treated with L-NAME or PBS as a control immediately after light exposure. Western blot analysis showed that cleaved caspase-3 expression was undetectable in the normal retina, increased obviously at 1 day after light exposure in the PBS-treated group, and declined significantly at 1 day after light exposure in the L-NAME-treated group (Figure 6A,C). Dexras1 expression was relatively low in the normal retina, increased obviously at 1 day after light exposure in the PBS-treated group, and declined significantly in the L-NAME-treated group (Figure 6B,D). Furthermore, immunofluorescence was performed to detect the colabeling of cleaved caspase-3 and Dexras1 decreased in the retina at 1 day after light exposure in the L-NAME-treated group (Figure 6H-J) compared with the PBS-treated group (Figure 6E-G).

## Discussion

In addition to photoreceptor cell damage, excessive light can result in degenerative alterations in the inner retina, as well as ganglion cell death [23-25]. Light has a direct effect on ganglion cell damage [7]. Given that nNOS participates in the pathological progress of RGC damage [14,26], we were interested in investigating whether Dexras1 contributes to RGC death in the light damage model.

In this report, we delineated that light-induced Dexras1 upregulated significantly at mRNA and protein expression in the retina. Changes in *Dexras1* mRNA and protein expression shared the similar temporal pattern that Dexras1 was constitutively expressed in the normal retina and reached the peak at 1 day after light exposure, gradually recovering to the baseline level at 7 days. If this is the case, the transient high expression of Dexras1 may provide a new insight into the molecular mechanism of retinal light damage.

Our experiment is the first to show the spatial distribution of Dexras1 in the retina by immunohistochemistry. These results demonstrated that Dexras1 immunoreactivity appeared in the GCL at a relatively low level. At 1 day after light exposure, Dexras1 expression increased significantly in the GCL. Furthermore, immunofluorescence revealed that the Dexras1 expression localized in RGCs was confirmed by NeuN. This indicates that Dexras1 may be involved in RGC damage after light exposure.

Caspases, which are important mediators of neuronal apoptosis, have been demonstrated to play a critical role in pathological cell death in the nervous system [27]. Caspase activation has been shown in retinal degeneration in several animal models [28,29]. In this study, Dexras1 colocalized with the apoptotic marker (cleaved caspase-3) in RGCs. These results showed that light-induced RGC apoptosis could be mediated by Dexras1.

Consensus indicates that exposure to excessive levels of light results in photoreceptor cells apoptosis [6,30,31]. Interestingly, we observed that cleaved caspase-3 immunoreactivity was undetectable in photoreceptor cells. However, we showed that ONL thickness decreased significantly after light exposure compared with the normal retina, which was consistent with previous studies [22,32,33]. Furthermore, TUNEL labeling showed that TUNEL-positive cells were present in the ONL, INL, and GCL. These data support the idea that photoreceptor cells also undergo apoptosis in this model. A possible explanation for this is that photoreceptor cells do not experience caspase-dependent apoptosis. In fact, the caspase-independent apoptotic pathway in photoreceptor cells has been well reported [13,34,35]. A steadily increasing number of reports suggest that not all cell types require caspase activity to undergo apoptosis [36,37]. These observations demonstrate that RGC apoptosis was caspase-dependent and photoreceptor cell apoptosis may be caspase-independent. The different mechanisms of cell death deserve further investigations.

nNOS plays a key role in the light-induced retinal degeneration model [13]. Dexras1 binds to nNOS via the adaptor protein CAPON, which is a 55 kDa protein that contains a C-terminal domain that binds to the PSD95/DLG/ZO-1 domain of nNOS [18]. The ternary complex of Dexras1, nNOS, and CAPON enables NO to S-nitrosylate NMDARs and alters their signaling [38]. In this report, the colocalization of Dexras1, nNOS, and CAPON was observed in RGCs, giving evidence of the existence of a ternary complex in the retina. These data show that Dexras1-mediated RGC apoptosis may be associated with nNOS.

Pharmacological inhibition of NOS activity by L-NAME has been investigated for its possible neuroprotective effect on RGCs [26,39]. Systemic treatment with NMDAR antagonists has a similar protective effect on RGCs compared with systemic treatment with L-NAME [40]. In the current study, intraperitoneal administration of L-NAME was monitored in the retina after light exposure. The results showed that L-NAME prevented RGCs apoptosis by decreasing cleaved caspase-3 and Dexras1 expression. These results further support the claim that Dexras1 participates in RGC apoptosis in conjunction with activating nNOS.

In conclusion, we demonstrated that the transient high expression of Dexras1 is localized in apoptotic RGCs and colocalized with nNOS and CAPON. The NOS inhibitor attenuated RGC apoptosis by reducing cleaved caspase-3 and Dexras1 expression, implicating that Dexras1-mediated RGC damage appeared in nNOS activation. Dexras1 is a novel Ras-like G protein that modulates multiple signaling cascades. Further investigations are necessary to explore the signal transduction of Dexras1 in RGC apoptosis.

## References

[1] Boulton M Rózanowska M Rózanowski B Retinal photodamage. J Photochem Photobiol B 2001 64 144 61 11744401 10.1016/s1011-1344(01)00227-5

[2] Noell WK Walker VS Kang BS Berman S Retinal damage by light in rats. Invest Ophthalmol 1966 5 450 73 5929286

[3] Chen L Wu W Dentchev T Wong R Dunaief JL Increased metallothionein in light damaged mouse retinas. Exp Eye Res 2004 79 287 93 15325575 10.1016/j.exer.2004.05.004

[4] Gordon WC Casey DM Lukiw WJ Bazan NG DNA damage and repair in light-induced photoreceptor degeneration. Invest Ophthalmol Vis Sci 2002 43 3511 21 12407163

[5] Marc RE Jones BW Watt CB Vazquez-Chona F Vaughan DK Organisciak DT Extreme retinal remodeling triggered by light damage: implications for age related macular degeneration. Mol Vis 2008 14 782 806 18483561 PMC2375357

[6] Wenzel A Grimm C Samardzija M Remé CE Molecular mechanisms of light-induced photoreceptor apoptosis and neuroprotection for retinal degeneration. Prog Retin Eye Res 2005 24 275 306 15610977 10.1016/j.preteyeres.2004.08.002

[7] Osborne NN Li GY Ji D Mortiboys HJ Jackson S Light affects mitochondria to cause apoptosis to cultured cells: possible relevance to ganglion cell death in certain optic neuropathies. J Neurochem 2008 105 2013 28 18315568 10.1111/j.1471-4159.2008.05320.x

[8] Wood JP Lascaratos G Bron AJ Osborne NN The influence of visible light exposure on cultured RGC-5 cells. Mol Vis 2007 14 334 44 18334950 PMC2254956

[9] Goldstein IM Ostwald P Roth S Nitric oxide: a review of its role in retinal function and disease. Vision Res 1996 36 2979 94 8917798 10.1016/0042-6989(96)00017-x

[10] Estévez AG Spear N Manuel SM Radi R Henderson CE Barbeito L Beckman JS Nitric oxide and superoxide contribute to motor neuron apoptosis induced by trophic factor deprivation. J Neurosci 1998 18 923 31 9437014 10.1523/JNEUROSCI.18-03-00923.1998 PMC6792767

[11] Michel T Feron O Nitric oxide synthases: which, where, how, and why? J Clin Invest 1997 100 2146 52 9410890 10.1172/JCI119750 PMC508408

[12] Nathan C Inducible nitric oxide synthase: what difference does it make? J Clin Invest 1997 100 2417 23 9366554 10.1172/JCI119782 PMC508440

[13] Donovan M Carmody RJ Cotter TG Light-induced photoreceptor apoptosis in vivo requires neuronal nitric-oxide synthase and guanylate cyclase activity and is caspase-3-independent. J Biol Chem 2001 276 23000 8 11278285 10.1074/jbc.M005359200

[14] Brown GC Borutaite V Nitric oxide inhibition of mitochondrial respiration and its role in cell death. Free Radic Biol Med 2002 33 1440 50 12446201 10.1016/s0891-5849(02)01112-7

[15] Carroll RC Zukin RS NMDA-receptor trafﬁcking and targeting: implications for synaptic transmission and plasticity. Trends Neurosci 2002 25 571 7 12392932 10.1016/s0166-2236(02)02272-5

[16] Cheah JH Kim SF Hester LD Clancy KW Patterson SE 3rd Papadopoulos V Snyder SH NMDA Receptor-nitric oxide transmission mediates neuronal iron homeostasis via the GTPase Dexras1. Neuron 2006 51 431 40 16908409 10.1016/j.neuron.2006.07.011 PMC3150500

[17] Cismowski MJ Takesono A Ma C Lizano JS Xie X Fuernkranz H Lanier SM Duzic E Genetic screens in yeast to identify mammalian nonreceptor modulators of G-protein signaling. Nat Biotechnol 1999 17 878 83 10471929 10.1038/12867

[18] Jaffrey SR Snowman AM Eliasson MJ Cohen NA Snyder SH CAPON: a protein associated with neuronal nitric oxide synthase that regulates its interactions with PSD95. Neuron 1998 20 115 24 9459447 10.1016/s0896-6273(00)80439-0

[19] Fang M Jaffrey SR Sawa A Ye K Luo X Snyder SH Dexras1: a G protein speciﬁcally coupled to neuronal nitric oxide synthase via CAPON. Neuron 2000 28 183 93 11086993 10.1016/s0896-6273(00)00095-7

[20] Li X Cheng C Fei M Gao S Niu S Chen M Liu Y Guo Z Wang H Zhao J Yu X Shen A Spatiotemporal expression of Dexras1 after spinal cord transection in rats. Cell Mol Neurobiol 2008 28 371 88 18219571 10.1007/s10571-007-9253-y PMC11515033

[21] Shen A Chen M Niu S Sun L Gao S Shi S Li X Lv Q Guo Z Cheng C Changes in mRNA for CAPON and Dexras1 in adult rat following sciatic nerve transaction. J Chem Neuroanat 2008 35 85 93 17768032 10.1016/j.jchemneu.2007.07.004

[22] Sun MH Pang JH Chen SL Kuo PC Chen KJ Kao LY Wu JY Lin KK Tsao YP Photoreceptor protection against light damage by AAV-mediated overexpression of heme oxygenase-1. Invest Ophthalmol Vis Sci 2007 48 5699 707 18055822 10.1167/iovs.07-0340

[23] Thanos S Heiduschka P Romann I Exposure to a solar eclipse causes neuronal death in the retina. Graefes Arch Clin Exp Ophthalmol 2001 239 794 800 11760043 10.1007/s004170100362

[24] Codenotti M Patelli F Brancato R OCT findings in patients with retinopathy after watching a solar eclipse. Ophthalmologica 2002 216 463 6 12569943 10.1159/000067540

[25] Iandiev I Wurm A Hollborn M Wiedemann P Grimm C Remé CE Reichenbach A Pannicke T Bringmann A Müller cell response to blue light injury of the rat retina. Invest Ophthalmol Vis Sci 2008 49 3559 67 18450590 10.1167/iovs.08-1723

[26] Park SH Kim JH Kim YH Park CK Expression of neuronal nitric oxide synthase in the retina of a rat model of chronic glaucoma. Vision Res 2007 47 2732 40 17825345 10.1016/j.visres.2007.07.011

[27] Springer JE Nottingham SA McEwen ML Azbill RD Jin Y Caspase-3 apoptotic signaling following injury to the central nervous system. Clin Chem Lab Med 2001 39 299 307 11388652 10.1515/CCLM.2001.046

[28] Katai N Yoshimura N Apoptotic retinal neuronal death by ischemia-reperfusion is executed by two distinct caspase family proteases. Invest Ophthalmol Vis Sci 1999 40 2697 705 10509668

[29] Kermer P Klöcker N Labes M Thomsen S Srinivasan A Bähr M Activation of caspase-3 in axotomized rat retinal ganglion cells in vivo. FEBS Lett 1999 453 361 4 10405176 10.1016/s0014-5793(99)00747-4

[30] Schmitz-Valckenberg S Guo L Cheung W Moss SE Fitzke FW Cordeiro MF In vivo imaging of retinal cell apoptosis following acute light exposure. Ophthalmologe 2010 107 22 9 19499229 10.1007/s00347-009-1952-y

[31] Organisciak DT Vaughan DK Retinal light damage: mechanisms and protection. Prog Retin Eye Res 2010 29 113 34 19951742 10.1016/j.preteyeres.2009.11.004 PMC2831109

[32] Tanito M Li F Elliott MH Dittmar M Anderson RE Protective effect of TEMPOL derivatives against light-induced retinal damage in rats. Invest Ophthalmol Vis Sci 2007 48 1900 5 17389526 10.1167/iovs.06-1057

[33] Read SP Cashman SM Kumar-Singh R POD nanoparticles expressing GDNF provide structural and functional rescue of light-induced retinal degeneration in an adult mouse. Mol Ther 2010 18 1917 26 20700110 10.1038/mt.2010.167 PMC2990513

[34] Chahory S Padron L Courtois Y Torriglia A The LEI/L-DNase II pathway is activated in light-induced retinal degeneration in rats. Neurosci Lett 2004 367 205 9 15331154 10.1016/j.neulet.2004.06.004

[35] Chahory S Keller N Martin E Omri B Crisanti P Torriglia A Light induced retinal degeneration activates a caspase-independent pathway involving cathepsin D. Neurochem Int 2010 57 278 87 20558223 10.1016/j.neuint.2010.06.006

[36] Okuno S Shimizu S Ito T Nomura M Hamada E Tsujimoto Y Matsuda H Bcl-2 prevents caspase-independent cell death. J Biol Chem 1998 273 34272 7 9852091 10.1074/jbc.273.51.34272

[37] Lavoie JN Nguyen M Marcellus RC Branton PE Shore GC E4orf4, a novel adenovirus death factor that induces p53-independent apoptosis by a pathway that is not inhibited by zVAD-fmk. J Cell Biol 1998 140 637 45 9456323 10.1083/jcb.140.3.637 PMC2140159

[38] Lipton SA Choi YB Pan ZH Lei SZ Chen HS Sucher NJ Loscalzo J Singel DJ Stamler JS A redox-based mechanism for the neuroprotective and neurodestructive effects of nitric oxide and related nitroso-compounds. Nature 1993 364 626 32 8394509 10.1038/364626a0

[39] Katsuki H Yamamoto R Nakata D Kume T Akaike A Neuronal nitric oxide synthase is crucial for ganglion cell death in rat retinal explant cultures. J Pharmacol Sci 2004 94 77 80 14745122 10.1254/jphs.94.77

[40] Nucci C Tartaglione R Rombolà L Morrone LA Fazzi E Bagetta G Neurochemical evidence to implicate elevated glutamate in the mechanisms of high intraocular pressure (IOP)-induced retinal ganglion cell death in rat. Neurotoxicology 2005 26 935 41 16126273 10.1016/j.neuro.2005.06.002

